# In Vitro Activities of LCB 01-0648, a Novel Oxazolidinone, against Gram-Positive Bacteria

**DOI:** 10.3390/molecules22030394

**Published:** 2017-03-03

**Authors:** Sang-Hun Oh, Josep Kim, Sung-Yoon Baek, Sang-Eun Chae, Hee-Soo Park, Young-Lag Cho, Jin-Hwan Kwak

**Affiliations:** 1 School of Life Science, Handong Global University, Pohang 37554, Korea; osh8755@naver.com (S.-H.O.); indy8152@naver.com (J.K.); 2 LegoChem BioSciences. Inc., Daejeon 34302, Korea; bsy@legochembio.com (S.-Y.B.); sangeun@legochembio.com (S.-E.C.); young@legochembio.com (Y.-L.C.); 3 School of Food Science and Biotechnology, Institute of Agricultural Science & Technology, Kyungpook National University, Daegu 41566, Korea; phsoo97@knu.ac.kr

**Keywords:** LCB01-0648, oxazolidinone, MICs, linezolid-resistant *S. aureus*

## Abstract

Oxazolidinones are a novel class of synthetic antibacterial agents that inhibit bacterial protein synthesis. Here, we synthesized and tested a series of oxazolidinone compounds containing cyclic amidrazone. Among these compounds, we further investigated the antibacterial activities of LCB01-0648 against drug-susceptible or resistant Gram-positive cocci in comparison with those of six reference compounds. LCB01-0648 showed the most potent antimicrobial activities against clinically isolated Gram-positive bacteria. Against the methicillin-resistant *Staphylococcus aureus* (MRSA) and methicillin-resistant coagulase-negative staphylococci (MRCNS) isolates, LCB01-0648 showed the lowest MIC_90_s (0.5 mg/L) among the tested compounds. In addition, LCB01-0648 had the lowest minimum inhibitory concentrations (MICs) against the four linezolid-resistant *S. aureus* (LRSA) strains (range 2–4 mg/L). The results of the time–kill studies demonstrated that LCB01-0648 at a concentration 8× the (MIC) showed bactericidal activity against methicillin-susceptible *Staphylococcus aureus* MSSA or MRSA, but showed a bacteriostatic effect against LRSA. These results indicate that LCB01-0648 could be a good antibacterial candidate against multidrug-resistant (MDR) Gram-positive cocci.

## 1. Introduction

The rapid increase in antibacterial-resistant Gram-positive bacteria including methicillin-resistant *Staphylococcus aureus* (MRSA), beta-lactam-resistant *Streptococcus pneumoniae*, and vancomycin-resistant enterococci (VRE) is a great concern for global health [1,2,3]. However, antibacterial-resistant pathogens that are resistant to most available antibiotics are currently a more pressing concern [4,5]. Therefore, novel antibacterial agents are required for the treatment of infectious disease caused by multidrug-resistant (MDR) Gram-positive pathogens [6,7].

Oxazolidinones are a novel group of synthetic antibiotics that mainly have antibacterial activities against Gram-positive organisms [8,9,10]. This class of antibiotics specifically binds to the 23S ribosomal RNA of the 50S ribosomal subunit, and thereby prevents the formation of the 70S ribosomal complex, a key component of the translation process in bacteria [9,11,12,13,14]. In 2000, linezolid was the first oxazolidinone antibiotic approved by the US Food and Drug Administration (FDA) for clinical use [15,16,17]. This oxazolidinone antibiotic has been used for the treatment of patients with skin and soft tissue infection and pneumonia caused by MDR Gram-positive pathogens [18,19,20,21]. However, it has been reported that several linezolid-resistant staphylococci have emerged worldwide [22,23,24]. These linezolid-resistant *S. aureus* (LRSA) strains have the 23S rRNA mutation or the Cfr ribosomal methyltransferase [22,25,26,27]. To overcome infection by LRSA strains, several research groups are developing analogs of oxazolidinones, which have potent activity against MDR Gram-positive cocci including LRSA and *Mycobacterium tuberculosis* [28,29,30,31]. Sutezolid, posizolid and ranbezolid are under development for the treatment of Gram-positive infections [32,33,34,35,36]. Recently, tedizolid phosphate (Sivextro) was approved by the FDA for the treatment of acute bacterial skin infection [37,38,39].

**Figure 1:**
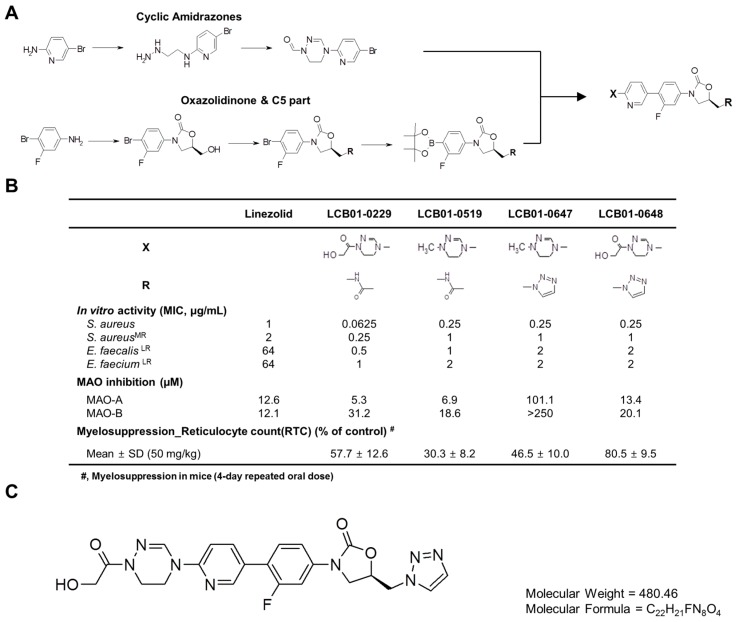
Chemical structure of LCB01-0648. (**A**) Synthetic scheme of oxazolidinones containing cyclic amidrazone; (**B**) Structure–activity relationship of cyclic amidrazone; (**C**) Structure of LCB01-0648.

Previously, we reported novel oxazolidinone compounds such as LCB01-0062 and LCB01-0371 which showed potent antibacterial activities against most Gram-positive cocci [40,41]. While LCB01-0371 has high potency and safety, this oxazolidinone showed moderate activity against linezolid-resistant strains [41]. To overcome this weakness, we modified the C-5 side change of the oxazolidinone A-ring (R) and the oxazolidinone C-ring (X) (Figure 1A). We synthesized a series of oxazolidinone compounds containing cyclic amidrazone and presented the structure–activity relationship of these oxazolidinone compounds which showed enhanced potencies against MRSA and linezolid-resistant VRE (Figure 1B). Among them, we selected LCB01-0648 (Figure 1C) as a lead compound for a further evaluation due to its high potent activity and low safety issues. In this study, we investigated the antibacterial activities of LCB01-0648 against clinically isolated drug-susceptible and -resistant Gram-positive cocci.

## 2. Results

As shown in Figure 1B, we synthesized four oxazolidinone compounds containing cyclic amidrazone, LCB01-0229, LCB01-0519, LCB01-0647, and LCB01-0648. We evaluated the in vitro activities of these four oxazolidinones against MRSA and linezolid-resistant VRE, and found that four oxazolidinone compounds have potent activities in test organisms. We then assessed the safety of these compounds using monoamine oxidase (MAO) inhibition and myelotoxicity. LCB01-0648 showed less bone marrow toxicity of LCB01-0648, lower than the toxicities of other compounds (data not shown). LCB01-0648 showed weak inhibition of MAO activity, with 13.4 and 20.1 μM for MAO-A and MAO-B, respectively. Due to the low bone marrow toxicity, we selected LCB01-0648 as a lead compound.

To further investigated the antibacterial activities of LCB01-0648, the MICs of LCB01-0648 against the 610 clinically isolated Gram-positive cocci were tested and compared with those of linezolid, oxacillin, erythromycin, ciprofloxacin, sparfloxacin, moxifloxacin, gemifloxacin, vancomycin, and quinupristin–dalfopristin. The MICs of LCB01-0648 against the clinically isolated staphylococci were 0.125–1 mg/L (Table 1). Against the MRSA and MRCNS isolates, LCB01-0648 showed the lowest MIC_90_s (0.5 mg/L) among the tested compounds. All staphylococci strains were also susceptible to linezolid (MICs 1 to 2 mg/L), quinupristin–dalfopristin (MICs 0.25 to 4 mg/L), and vancomycin (MICs 0.125 to 2 mg/L).

Table 2 showed the antibacterial activity of LCB01-0648 against streptococci in comparison with those of reference compounds. The MIC90s of LCB01-0648 against *S. pneumoniae* and *Streptococcus pyogenes* were 0.25 and 0.25 mg/L, respectively. LCB01-0648 showed the most potent antibacterial activity against the clinically isolated streptococci and was four to eight times more active than linezolid was.

Against enterococci, LCB01-0648 showed the most potent activity among the tested compounds (Table 3). MIC_90_s of LCB01-0648 against *Enterococcus faecalis* and *Enterococcus faecium* were 0.5 and 0.5 mg/L, respectively. Against 47 VRE, LCB01-0648 had the lowest MIC_90_ (0.5 mg/L), followed by linezolid (MIC_90_ 2 mg/L), quinupristin–dalfopristin (MIC_90_ 2 mg/L), gemifloxacin (MIC_90_, 32 mg/L), moxifloxacin (MIC_90_, 32 mg/L), sparfloxacin (MIC_90_, 64 mg/L), ciprofloxacin (MIC_90_, >64 mg/L).

To evaluate the antimicrobial activity of LCB01-0648 against the LRSA, we evaluated the MICs of LCB01-0648 and the other compounds against four LRSA strains [42,43,44]. LCB01-0648 had the lowest MICs against the four LRSA strains (range 2–4 mg/L), followed by moxifloxacin (4–64 mg/L), gemifloxacin (8–64 mg/L), linezolid (8–64 mg/L), sparfloxacin (16–64 mg/L), oxacillin (range 32–64 mg/L), and ciprofloxacin (>64 mg/L) (Table 4). These results demonstrate that LCB01-0648 is a potent compound against MDR staphylococci.

**Figure 2:**
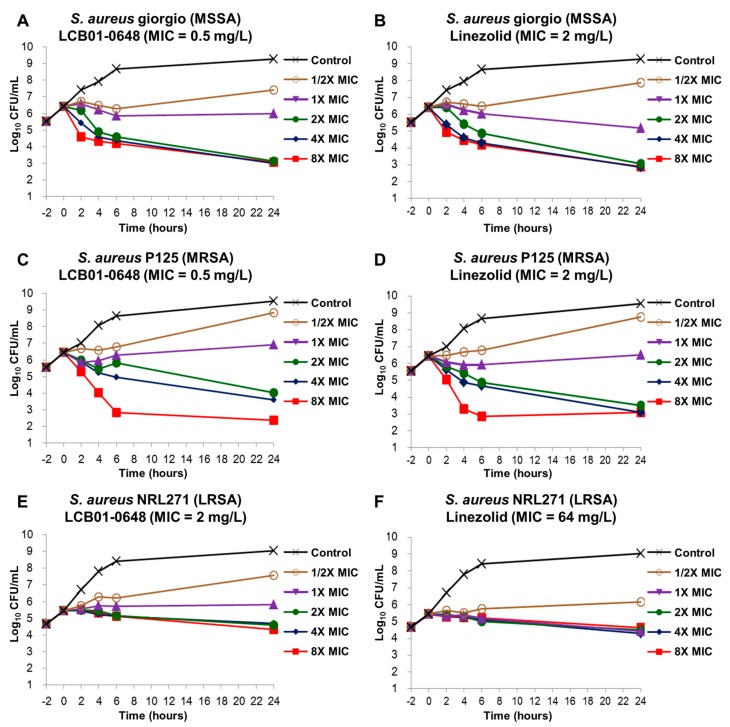
Time–kill curves of LCB01-0648 and linezolid against *Staphylococcus aureus*. *S. aureus* Giorgio (methicillin-susceptible *S. aureus*, MSSA) exposed to (**A**) LCB01-0648 or (**B**) linezolid (**B**). *S. aureus* P125 (methicillin-resistant *S. aureus*, MRSA) exposed to (**C**) LCB01-0648 or (**D**) or linezolid. (**E** and **F**) *S. aureus* NRS271 (linezolid-resistant *S. aureus*, LRSA) exposed to LCB01-0648.

To determine whether LCB01-0648 has bactericidal or bacteriostatic activity, we carried out an in vitro time–kill assay with LCB01-0648 and linezolid against the methicillin-susceptible *S. aureus* (MSSA) Giorgio, MRSA P125, and LRSA NRS271. As shown in Figure 2, LCB01-0648 and linezolid had bactericidal activity at a concentration 4× the MIC against the MSSA and MRSA. LCB01-0648 and linezolid had bacteriostatic activity against the LRSA at a concentration 8× the MICs after 24-h incubation (Figure 2E,F). However, we did not observe any re-growth at a concentration 8× the MICs.

## 3. Discussion

The oxazolidinone compounds are narrow-spectrum agents that have antibacterial activity against Gram-positive cocci [10]. Linezolid was the only oxazolidinone antibiotic approved by the FDA until 2014 [17]. Recently, tedizolid has been used to treat acute skin infections caused by Gram-positive cocci [37,38,39]. In this study, we investigated the in vitro activity of a novel oxazolidinone, LCB01-0648. LCB01-0648 was four- to eight-fold more active than linezolid was against drug-susceptible and -resistant staphylococci, streptococci, and enterococci, including MRSA and VRE (Table 1, Table 2 and Table 3). Importantly, LCB01-0648 also exhibited potent antibacterial activity against linezolid-LRSA strains that carried the G2576U mutation of the 23S rRNA (*S. aureus* NRS119, NRS121, and NRS271) or the ribosomal protein L3 RplC (*S. aureus* NRS127) [43] (Table 4). These results suggest that LCB01-0648 might be a second-generation oxazolidinone derivative. Our time–kill results showed that LCB01-0648 had bacteriostatic activity against drug-susceptible and resistant *S. aureus* (Figure 2).

Previously, we synthesized novel oxazolidinones including LCB01-0062 and LCB01-0371 [40,41]. These oxazolidinones showed potent in vitro and in vivo activities against most Gram-positive cocci. However, LCB01-0371 showed moderate activity against linezolid-resistant *S. aureus.* A series of studies demonstrated that the CD-rings of certain oxazolidinones play an important role for interaction between oxazolidinone and the peptidyl transferase center binding site [45,46,47]. In addition, either hydroxymethyl or 1,2,3-triazole C-5 groups are crucial for antibacterial activity against cfr strains [29,36]. We assumed that LCB01-0371 might show lower potency against linezolid-resistant *S. aureus* or cfr strains due to the lack of a D-ring substitute. To overcome this, we synthesized a series of novel oxazolidinone compounds containing cyclic amidrazone at the D-ring. These compounds containing cyclic amidrazone showed enhanced activities against linezolid resistant Gram-positive cocci. Among them, LCB01-0648 showed strong activity against tested organisms and low toxicity. Therefore, LCB01-0648 was selected as a lead compound for a further evaluation. In this study, we focused on the in vitro antibacterial activities against clinically isolated Gram-positive cocci. In order to develop LCB01-0648 as a novel second-generation oxazolidinone candidate, in vivo activities of LCB01-0648 should be examined in various mouse models. In addition, pharmacokinetics and pharmacodynamics studies for LCB01-0648 should be assessed.

## 4. Materials and Methods

### 4.1. Antimicrobial Agents

LCB01-0648 (purity, 99%; analyzed by HPLC) and linezolid were synthesized at LegoChem Biosciences, Inc., Daejeon, Korea. Oxacillin, vancomycin, and erythromycin were obtained from Sigma-Aldrich, Seoul, Korea. Ciprofloxacin, moxifloxacin, and sparfloxacin were provided by the R & D Center, Dong-Wha Pharmaceutical Industry Co., Ltd., Anyang, Korea. Gemifloxacin was obtained from LG Chemical Ltd. (Daejeon, Korea). Quinupristin-dalporistin was obtained from CrystalGenomics Inc. (Seongnam, Korea).

### 4.2. Bacterial Strains

For the antibiotic susceptibility testing, the tested Gram-positive cocci were isolated from human clinical specimens that were obtained from several hospitals in Seoul, Korea, from 2006 to 2015. A total of 610 Gram-positive cocci strains including 326 staphylococci, 100 streptococci, and 184 enterococci strains were used in this study. Four LRSA strains, *S. aureus* NRS119, NRS121, NRS127, and NRS271 were obtained from LegoChem Biosciences, Inc., while the *S. aureus* Giorgio was obtained from LG Chemical Ltd. (Daejeon, Korea) and was used for the time–kill assay.

### 4.3. Susceptibility Test

To determine the MICs of the aerobic organisms, a two-fold agar dilution method was carried out as described by the Clinical and Laboratory Standards Institute (CLSI) [48]. The test strains were grown for 18 h at 37 °C in Mueller Hinton Agar (MHA, Difco, Sparks, MD, USA) and subcultured in Mueller Hinton II Broth (MHIIB, Difco) except for the *Streptococci*. Bacteria were incubated for 18 h at 37 °C and diluted with the same fresh medium to a density of approximately 10^6^ CFU/mL. *S. pneumoniae* and *S. pyogenes* were grown on MHA supplemented with 5% defibrinated sheep blood (Komed, Seongnam Korea). The diluted organisms were transferred to the wells of a multipin inoculator and were inoculated onto the drug-containing agar plates. The plates were incubated at 37 °C for 24 h and were checked for growth. The MIC was determined to be the lowest concentration that completely inhibited growth on the agar plates, disregarding a single colony or a faint haze caused by the inoculum.

### 4.4. Time–Kill Assay

The time–kill studies were performed using the CLSI M26-A method [49]. The test microbes were cultured for 18 h at 37 °C in MHIIB and were diluted with fresh MHIIB to a density of 10^5^ to 10^6^ CFU/mL. The diluted cultures were pre-incubated for 2 h, and then the LCB01-0648 or linezolid was added to the cultures at concentrations of 0.5×, 1×, 2×, 4×, and 8× MIC. The numbers of viable cells were quantified after 0-, 2-, 4, 6-, 24-h incubation at 37 °C for 18 h by serial dilution on MHA. The compounds were considered bactericidal at the concentration that reduced the original inoculum by 3 log of CFU/mL (99.9%) at each of the time periods. Alternatively, the compounds were considered bacteriostatic if the inoculum was reduced by 0–3 log of CFU/mL.

### 4.5. Monoamine Oxidase Inhibition Assays

Test compounds (Linerzolid, LCB01-0229, LCB01-0519, LCB01-0647, and LCB01-0648) were dissolved in DMSO at a concentration of 50 mM and diluted to 4-fold concentration of the final concentration with enzyme reaction buffer. MAO-A and MAO-B were purchased from Sigma (St. Louis, MO, USA). MAO-Glo^TM^ Assay Kit was purchased from Promega (Madison, WI, USA). An enzyme assay was performed according to MAO-Glo^TM^ Assay Kit protocol. The IC_50_ value was calculated by the nonlinear regression method using GraFit software (Erithacus, London, UK). IC_50_ refers to the concentration of compound that inhibits a reaction by 50% for MAO-A or MAO-B compared to the negative control.

### 4.6. Myelosuppression Assay

A myelosuppression assay was carried out as previously described with modification [50]. Animal protocols were approved by the Institutional Animal Care and Use Committee. Six or seven weeks old female C3H mice were purchased from Orient Bio. Charles River Laboratories (Seongnam). Mice were maintained in 22 ± 3 °C with 50% ± 20% relative humidity with water and diet (5L79, orient bio) and housed in polycarbonate cages. Individual dose (50 mg/kg) was calculated for each animal based on the most recently recorded body weight with the dose volume of 10 mL/kg. The animals were orally administered using a sonde attached to disposable syringes. Dosing formulation was administered once daily for 4 days. Mice were sacrificed by ether 4 days after treatment. All animals had been fasted for approximately 16 h prior to sacrifice. Blood samples were collected from the vena cava and were taken into a tube containing EDTA (Ethylenediaminetetraacetic acid) (Becton Dickinson, Sparks, MD, USA). Samples were analyzed by Biotoxtech Co. Ltd. (Cheongju, South Korea).

## 5. Conclusions

Our in vitro results demonstrate that LCB01-0648 had potent antibacterial activities against MDR Gram-positive pathogens including MRSA, LRSA, and VRE. Therefore, LCB01-0648 is worth further evaluation as a new second-generation oxazolidinone candidate that will be available for the clinical treatment of serious skin infections caused by multi-drug Gram-positive cocci including LRSA.

## Figures and Tables

**Table 1:** Minimum inhibitory concentrations (MICs) of LCB01-0648, linezolid, oxacillin, erythromycin, ciprofloxacin, sparfloxacin, moxifloxacin, gemifloxacin, vancomycin, and quinupristin–dalfopristin for clinical isolates of Staphylococci.

Antimicrobial Agents	MSSA (*n* = 74) ^a^			MRSA (*n* = 200) ^a^			MSCNS (*n* = 19) ^a^			MRCNS (*n* = 33) ^a^		
	Range	MIC_50_	MIC_90_	Range	MIC_50_	MIC_90_	Range	MIC_50_	MIC_90_	Range	MIC_50_	MIC_90_
LCB01-0648	0.25–0.5	0.5	0.5	0.125–0.5	0.5	0.5	0.125–0.5	0.25	0.5	0.125–1	0.25	0.5
Linezolid	2	2	2	1–2	2	2	1–2	1	2	1–2	1	2
Oxacillin	0.06–4	0.25	0.5	8–64	>64	>64	0.03–1	0.125	1	2–64	>64	>64
Erythromycin	0.125–64	0.25	>64	0.25–64	>64	>64	0.06–64	0.25	>64	0.06–64	>64	>64
Ciprofloxacin	0.06–64	0.25	0.5	0.125–64	32	>64	0.06–8	0.125	8	0.06–64	8	32
Sparfloxacin	0.015–8	0.06	0.125	0.06–64	16	>64	0.03–8	0.125	4	0.03–32	4	16
Moxifloxacin	0.015–8	0.06	0.125	0.03–64	4	64	0.03–4	0.125	4	0.06–16	2	8
Gemifloxacin	0.008–8	0.015	0.06	0.008–64	2	64	0.008–0.5	0.015	0.5	0.008–8	0.5	4
Vancomycin	0.25–2	1	1	0.5–4	1	2	1–4	2	4	1–4	2	4
Quinupristin–dalfopristin	0.125–0.5	0.25	0.5	0.125–1	0.5	1	0.125–1	0.25	1	0.125–8	0.25	2

^a^ MSSA, methicillin-susceptible *Staphylococcus aureus*; MRSA, methicillin-resistant *S. aureus*; MSCNS, methicillin-susceptible coagulase-negative staphylococci; MRCNS, methicillin-resistant coagulase-negative *staphylococci*.

**Table 2:** Minimum inhibitory concentrations (MICs) of LCB01-0648, linezolid, oxacillin, erythromycin, ciprofloxacin, sparfloxacin, moxifloxacin, gemifloxacin, vancomycin, and quinupristin–dalfopristin for clinical isolates of Streptococci.

Antimicrobial Agents	*S. pneumoniae* (*n* = 79)			*S. pyogenes* (*n* = 21)		
	Range	MIC_50_	MIC_90_	Range	MIC_50_	MIC_90_
LCB01-0648	0.03–1	0.125	0.25	0.125–0.5	0.25	0.25
Linezolid	0.5–1	1	1	1–2	2	2
Oxacillin	0.008–32	16	16	0.25–32	0.5	8
Erythromycin	0.008–64	>64	>64	0.008–8	0.06	2
Ciprofloxacin	0.5–32	2	4	0.5–4	1	2
Sparfloxacin	0.06–16	0.25	0.5	0.125–1	0.25	0.5
Moxifloxacin	0.06–4	0.25	0.5	0.125–0.5	0.125	0.25
Gemifloxacin	0.008–0.25	0.03	0.06	0.03–0.125	0.03	0.06
Vancomycin	0.5–2	1	1	0.5–4	1	1
Quinupristin–dalfopristin	0.5–4	1	2	1–2	1	2

**Table 3:** Minimum inhibitory concentrations (MICs) of LCB01-0648, linezolid, oxacillin, erythromycin, ciprofloxacin, sparfloxacin, moxifloxacin, gemifloxacin, vancomycin, and quinupristin–dalfopristin for clinical isolates of Enterococci.

Antimicrobial Agents	*E. faecalis* (*n* = 108)			*E. faecium* (*n* = 29)			VRE (*n* = 47) ^a^		
	Range	MIC_50_	MIC_90_	Range	MIC_50_	MIC_90_	Range	MIC_50_	MIC_90_
LCB01-0648	0.125–0.5	0.25	0.5	0.25–0.5	0.25	0.5	0.125–0.5	0.25	0.25
Linezolid	1–2	2	2	1–2	2	2	1–2	2	2
Oxacillin	8–64	16	>64	16–64	>64	>64	>64–64	>64	>64
Erythromycin	0.125–64	>64	>64	0.125–64	>64	>64	>64–64	>64	>64
Ciprofloxacin	0.06–64	2	64	1~64	4	64	0.5–64	64	>64
Sparfloxacin	0.25–64	1	32	0.5–32	4	32	0.25–64	32	64
Moxifloxacin	0.06–64	1	32	0.25–64	4	32	0.25–32	16	32
Gemifloxacin	0.008–16	0.125	4	0.03–64	2	16	0.015–32	16	32
Vancomycin	0.5–4	2	4	0.5–8	1	2	>64–64	>64	>64
Quinupristin–dalfopristin	0.25–16	4	16	0.25–32	0.5	4	0.25–4	0.5	2

^a^ VRE, vancomycin-resistant enterococci.

**Table 4:** MIC of LCB01-0648, linezolid, oxacillin, ciprofloxacin, moxifloxacin and gemifloxacin against linezolid-resistant *S. aureus* strains.

Strains	Mutation Site	LCB01-0648	Linezolid	Oxacillin	Ciprofloxacin	Moxifloxacin	Gemifloxacin
*S. aureus* NRS119	G2576U	4	64	>64	>64	4	8
*S. aureus* NRS121	G2576U	4	64	>64	>64	4	8
*S. aureus* NRS127	Non-23s rRNA	2	8	32	>64	64	64
*S. aureus* NRS271	G2576U	2	64	>64	>64	16	16

## References

[1] Levy S.B., Marshall B. (2004). Antibacterial resistance worldwide: Causes, challenges and responses. Nat. Med..

[2] Lode H.M. (2009). Clinical impact of antibiotic-resistant gram-positive pathogens. Clin. Microbiol. Infect..

[3] Rice L.B. (2006). Antimicrobial resistance in gram-positive bacteria. Am. J. Infect. Control.

[4] Cornaglia G. (2009). Fighting infections due to multidrug-resistant gram-positive pathogens. Clin. Microbiol. Infect..

[5] Ventola C.L. (2015). The antibiotic resistance crisis: Part 1: Causes and threats. Pharm. Ther..

[6] Brown E.D., Wright G.D. (2016). Antibacterial drug discovery in the resistance era. Nature.

[7] Penesyan A., Gillings M., Paulsen I.T. (2015). Antibiotic discovery: Combatting bacterial resistance in cells and in biofilm communities. Molecules.

[8] Bozdogan B., Appelbaum P.C. (2004). Oxazolidinones: Activity, mode of action, and mechanism of resistance. Int. J. Antimicrob. Agents.

[9] Barbachyn M.R., Ford C.W. (2003). Oxazolidinone structure-activity relationships leading to linezolid. Angew. Chem. Int. Ed. Engl..

[10] Diekema D.J., Jones R.N. (2001). Oxazolidinone antibiotics. Lancet.

[11] Pandit N., Singla R.K., Shrivastava B. (2012). Current updates on oxazolidinone and its significance. Int. J. Med. Chem..

[12] Lin A.H., Murray R.W., Vidmar T.J., Marotti K.R. (1997). The oxazolidinone eperezolid binds to the 50s ribosomal subunit and competes with binding of chloramphenicol and lincomycin. Antimicrob. Agents Chemother..

[13] Patel U., Yan Y.P., Hobbs F.W., Kaczmarczyk J., Slee A.M., Pompliano D.L., Kurilla M.G., Bobkova E.V. (2001). Oxazolidinones mechanism of action: Inhibition of the first peptide bond formation. J. Biol. Chem..

[14] Wilson D.N., Schluenzen F., Harms J.M., Starosta A.L., Connell S.R., Fucini P. (2008). The oxazolidinone antibiotics perturb the ribosomal peptidyl-transferase center and effect trna positioning. Proc. Natl. Acad. Sci. USA.

[15] Perry C.M., Jarvis B. (2001). Linezolid: A review of its use in the management of serious gram-positive infections. Drugs.

[16] Moellering R.C. (2003). Linezolid: The first oxazolidinone antimicrobial. Ann. Intern. Med..

[17] Brickner S.J., Barbachyn M.R., Hutchinson D.K., Manninen P.R. (2008). Linezolid (zyvox), the first member of a completely new class of antibacterial agents for treatment of serious gram-positive infections. J. Med. Chem..

[18] Stevens D.L., Dotter B., Madaras-Kelly K. (2004). A review of linezolid: The first oxazolidinone antibiotic. Expert Rev. Anti-Infect. Ther..

[19] Bassetti M., Righi E., Di Biagio A., Rosso R., Beltrame A., Bassetti D. (2005). Role of linezolid in the treatment of orthopedic infections. Expert Rev. Anti-Infect. Ther..

[20] Bassetti M., Vitale F., Melica G., Righi E., Di Biagio A., Molfetta L., Pipino F., Cruciani M., Bassetti D. (2005). Linezolid in the treatment of gram-positive prosthetic joint infections. J. Antimicrob. Chemother..

[21] Chien J.W., Kucia M.L., Salata R.A. (2000). Use of linezolid, an oxazolidinone, in the treatment of multidrug-resistant gram-positive bacterial infections. Clin. Infect. Dis..

[22] Gu B., Kelesidis T., Tsiodras S., Hindler J., Humphries R.M. (2013). The emerging problem of linezolid-resistant staphylococcus. J. Antimicrob. Chemother..

[23] Sanchez Garcia M., De la Torre M.A., Morales G., Pelaez B., Tolon M.J., Domingo S., Candel F.J., Andrade R., Arribi A., Garcia N. (2010). Clinical outbreak of linezolid-resistant *Staphylococcus aureus* in an intensive care unit. JAMA.

[24] Stefani S., Bongiorno D., Mongelli G., Campanile F. (2010). Linezolid resistance in staphylococci. Pharmaceuticals.

[25] Tian Y., Li T., Zhu Y., Wang B., Zou X., Li M. (2014). Mechanisms of linezolid resistance in staphylococci and enterococci isolated from two teaching hospitals in shanghai, china. BMC Microbiol..

[26] Roman F., Roldan C., Trincado P., Ballesteros C., Carazo C., Vindel A. (2013). Detection of linezolid-resistant *Staphylococcus aureus* with 23s rRNA and novel l4 riboprotein mutations in a cystic fibrosis patient in Spain. Antimicrob. Agents Chemother..

[27] McCusker K.P., Fujimori D.G. (2012). The chemistry of peptidyltransferase center-targeted antibiotics: Enzymatic resistance and approaches to countering resistance. ACS Chem. Biol..

[28] Michalska K., Karpiuk I., Krol M., Tyski S. (2013). Recent development of potent analogues of oxazolidinone antibacterial agents. Bioorg. Med. Chem..

[29] Bhattarai D., Lee J.H., Seo S.H., Nam G., Choo H., Kang S.B., Kwak J.H., Oh T., Cho S.N., Pae A.N. (2014). Synthesis and in vitro evaluation of the antitubercular and antibacterial activity of novel oxazolidinones bearing octahydrocyclopenta[c]pyrrol-2-yl moieties. Chem. Pharm. Bull..

[30] Cynamon M.H., Klemens S.P., Sharpe C.A., Chase S. (1999). Activities of several novel oxazolidinones against *Mycobacterium tuberculosis* in a murine model. Antimicrob. Agents Chemother..

[31] Zhang M., Sala C., Dhar N., Vocat A., Sambandamurthy V.K., Sharma S., Marriner G., Balasubramanian V., Cole S.T. (2014). In vitro and in vivo activities of three oxazolidinones against nonreplicating *Mycobacterium tuberculosis*. Antimicrob. Agents Chemother..

[32] Sbardella G., Mai A., Artico M., Loddo R., Setzu M.G., La Colla P. (2004). Synthesis and in vitro antimycobacterial activity of novel 3-(*H*-pyrrol-1-yl)-2-oxazolidinone analogues of pnu-100480. Bioorg. Med. Chem. Lett..

[33] Williams K.N., Stover C.K., Zhu T., Tasneen R., Tyagi S., Grosset J.H., Nuermberger E. (2009). Promising antituberculosis activity of the oxazolidinone pnu-100480 relative to that of linezolid in a murine model. Antimicrob. Agents Chemother..

[34] Wookey A., Turner P.J., Greenhalgh J.M., Eastwood M., Clarke J., Sefton C. (2004). Azd2563, a novel oxazolidinone: Definition of antibacterial spectrum, assessment of bactericidal potential and the impact of miscellaneous factors on activity in vitro. Clin. Microbiol. Infect..

[35] Das B., Rudra S., Yadav A., Ray A., Rao A.V., Srinivas A.S., Soni A., Saini S., Shukla S., Pandya M. (2005). Synthesis and sar of novel oxazolidinones: Discovery of ranbezolid. Bioorg. Med. Chem. Lett..

[36] Kalia V., Miglani R., Purnapatre K.P., Mathur T., Singhal S., Khan S., Voleti S.R., Upadhyay D.J., Saini K.S., Rattan A. (2009). Mode of action of ranbezolid against staphylococci and structural modeling studies of its interaction with ribosomes. Antimicrob. Agents Chemother..

[37] Wong E., Rab S. (2014). Tedizolid phosphate (sivextro): A second-generation oxazolidinone to treat acute bacterial skin and skin structure infections. Pharm. Ther..

[38] Zhanel G.G., Love R., Adam H., Golden A., Zelenitsky S., Schweizer F., Gorityala B., Lagace-Wiens P.R., Rubinstein E., Walkty A. (2015). Tedizolid: A novel oxazolidinone with potent activity against multidrug-resistant gram-positive pathogens. Drugs.

[39] Burdette S.D., Trotman R. (2015). Tedizolid: The first once-daily oxazolidinone class antibiotic. Clin. Infect. Dis..

[40] Jung S.J., Yun I.N., Park H.S., Lee H.H., Jeong J.W., Kim Y.Z., Cho Y.L., Kwak J.H. (2012). Antibacterial activity of lcb01-0062, a novel oxazolidinone. Int. J. Antimicrob. Agents.

[41] Jeong J.W., Jung S.J., Lee H.H., Kim Y.Z., Park T.K., Cho Y.L., Chae S.E., Baek S.Y., Woo S.H., Lee H.S. (2010). In vitro and in vivo activities of lcb01-0371, a new oxazolidinone. Antimicrob. Agents Chemother..

[42] Miller K., Dunsmore C.J., Fishwick C.W., Chopra I. (2008). Linezolid and tiamulin cross-resistance in *Staphylococcus aureus* mediated by point mutations in the peptidyl transferase center. Antimicrob. Agents Chemother..

[43] Locke J.B., Hilgers M., Shaw K.J. (2009). Mutations in ribosomal protein l3 are associated with oxazolidinone resistance in staphylococci of clinical origin. Antimicrob. Agents Chemother..

[44] Shaw K.J., Poppe S., Schaadt R., Brown-Driver V., Finn J., Pillar C.M., Shinabarger D., Zurenko G. (2008). In vitro activity of tr-700, the antibacterial moiety of the prodrug tr-701, against linezolid-resistant strains. Antimicrob. Agents Chemother..

[45] Locke J.B., Finn J., Hilgers M., Morales G., Rahawi S., Kedar G.C., Picazo J.J., Im W., Shaw K.J., Stein J.L. (2010). Structure-activity relationships of diverse oxazolidinones for linezolid-resistant *Staphylococcus aureus* strains possessing the cfr methyltransferase gene or ribosomal mutations. Antimicrob. Agents Chemother..

[46] Poel T.J., Thomas R.C., Adams W.J., Aristoff P.A., Barbachyn M.R., Boyer F.E., Brieland J., Brideau R., Brodfuehrer J., Brown A.P. (2007). Antibacterial oxazolidinones possessing a novel *c*-5 side chain. (5r)-*trans*-3-[3-fluoro-4-(1-oxotetrahydrothiopyran-4-yl)phenyl]-2-oxooxazolidine-5-carboxylic acid amide (pf-00422602), a new lead compound. J. Med. Chem..

[47] Choy A.L., Vara Prasad J.V., Boyer F.E., Huband M.D., Dermyer M.R. (2007). Synthesis and sar of novel conformationally restricted oxazolidinones possessing gram-positive and fastidious gram-negative antibacterial activity. Part 2: Amino substitutions on heterocyclic d-ring system. Bioorg. Med. Chem. Lett..

[48] Clinical and Laboratory Standards Institute (2013). Performance Standards for Antimicrobial Susceptibility Testing. Twenty-Third Informational Supplement m100-s23.

[49] Clinical and Laboratory Standards Institute (1999). Methods for Determining Bactericidal Activity of Antimicrobial Agents: Approved Guideline m26-a.

[50] Hickey E.J., Gill C.J., Misura A.S., Flattery A.F., Abruzzo G.K. (2006). Experimental model of reversible myelosuppression caused by short-term, high-dose oxazolidinone administration. Therapy.

